# Histopathologic patterns of female genital tuberculosis with clinical correlation: a 10-year (2013–2022) retrospective cross-sectional study

**DOI:** 10.1186/s12905-024-03207-8

**Published:** 2024-06-26

**Authors:** Kidest Melkamu, Amanuel Damie, Senait Ashenafi, Moti Sori, Selfu Girma, Sofia Yimam, Negash Baye, Belachew Shote

**Affiliations:** 1 Department of Pathology, St. Peter’s Hospital, Addis Ababa, Ethiopia; 2 Department of Pathology, College of Health Sciences, Addis Ababa University, Addis Ababa, Ethiopia; 3 Armauer Hansen Research Institute, Addis Ababa, Ethiopia; 4 Department of Gynecology, Wolaita Sodo University, Wolaita Sodo, Ethiopia

**Keywords:** Extrapulmonary, Female genital, Infertility, Tuberculosis

## Abstract

**Objective:**

Underdiagnosis of female genital tuberculosis (FGTB) often leads to infertility. In this study, we aimed to determine the site and histopathologic patterns of FGTB and its correlation with clinical presentation and acid-fast bacilli (AFB) status.

**Methods:**

A retrospective cross-sectional study was conducted on 122 patients with a histopathological diagnosis of FGTB at the Department of Pathology, College of Health Sciences (CHS), Tikur Anbessa Specialized Hospital (TASH), Addis Ababa University (AAU), from January 1, 2013, to August 30, 2022.

**Results:**

Female genital tuberculosis was found in 0.94% of the gynecology specimens examined. The most common presentations were menstrual disturbance, abdominopelvic pain, and infertility. Among patients with FGTB, 4.6% exhibited misleading clinical and radiologic findings, leading to suspicion of malignancy and subsequent aggressive surgical management. The endometrium was the most frequently affected organ, followed by the fallopian tube, ovary, cervix, and vulva. In the majority of tuberculous endometritis cases (53.3%), histopathology revealed early-stage granulomas. Acid-fast bacilli were found in a significant proportion (42.6%) of FGTB tissues with TB histopathology. The ovary had the highest rate of AFB detection, followed by the fallopian tube, endometrium, and cervix.

**Conclusion:**

Female genital tuberculosis should be considered in reproductive-age women presenting with menstrual irregularities, abdominopelvic pain, infertility, or an abdominopelvic mass. The endometrium is commonly affected, displaying early granulomas with low AFB positivity.

## Introduction

Tuberculosis continues to be one of the leading causes of morbidity and mortality in low- and middle-income countries, despite the availability of effective diagnostic and therapeutic measures [1]. According to the World Health Organization (WHO), in 2020, an estimated 10 million people globally fell ill, resulting in 1.5 million deaths [2]. The 30 high TB-burden countries accounted for 86% of new TB cases [2]. Ethiopia is among these high TB burden countries, with an annual estimated incidence of 132 per 100,000 population and a death rate of 17 per 100,000 population in 2020 [2].

Genitourinary tuberculosis, which accounts for approximately 30% of all TB cases, is a common form of extrapulmonary tuberculosis (EPTB), and its prevalence is closely related to the overall prevalence of pulmonary TB in a community [3]. However, the burden of genital TB (GTB) in females is underestimated because most patients are asymptomatic. Apart from the disease’s subtle presentation, its magnitude is underreported due to the difficulty of diagnosis, which requires invasive techniques [4].

Female genital tuberculosis is an important cause of significant morbidity and long-term sequelae, especially infertility [5]. Conception rates remain low among infertile women with GTB even after multidrug therapy for TB [6]. In addition to infertility, it can lead to several sequelae, including chronic pelvic pain, ectopic pregnancy, miscarriage, and pelvic adhesions [7]. Early detection of FGTB is crucial for effective treatment and prevention of complications [7].

However, diagnosing FGTB can be challenging, as the disease often remains silent, with nonspecific and misleading symptoms [8]. As a result, it requires a high level of suspicion and the use of a combination of diagnostic tools. While imaging techniques can assist in the diagnosis of FGTB, they are not specific [8]. The definitive diagnosis of TB must rely on isolating tubercle bacilli, including AFB staining, bacterial culture, and PCR for *Mycobacteriumtuberculosis*, or histologic findings of granulomatous inflammation, particularly with caseation [8].

In our country, there is a lack of recent studies on the prevalence and histologic characteristics of FGTB, as well as its correlation to clinical characteristics. The most recent study was conducted in 1999, focusing on trends in female genital tract tuberculosis over twenty years. It examined the distribution of sites and clinical presentations of patients with genital TB. The study reported a progressive increase in the number of detected cases over the 20-year study period [9]. Given the prevalence of TB and human immunodeficiency virus (HIV) in Ethiopia, along with the recent disruptions in TB and HIV services caused by the global coronavirus disease (COVID-19) pandemic, there may be a significant increase in TB morbidity and mortality. Moreover, with more women seeking infertility treatment, the prevalence of FGTB is expected to rise.

Furthermore, it is expected that the age group commonly affected by FGTB, along with their clinical presentations, will change as the sociodemographic status of a population evolves [10]. In this research, we have examined the trend of FGTB over the past ten years, as well as the various histologic patterns of FGTB in different parts of the female genital tract, and their correlation with clinical presentations. Recognizing common histologic patterns will enhance the early diagnosis and management of patients, minimizing the need for unnecessary procedures. Therefore, this study will provide valuable insights for pathologists, clinicians, and researchers involved in the diagnosis and treatment of patients with FGTB.

## Methodology

### Study site and duration

A retrospective cross-sectional study was conducted at the Department of Pathology, College of Health Sciences (CHS), Tikur Anbessa Specialized Hospital (TASH), Addis Ababa University (AAU), Ethiopia. The study covered the period from January 1, 2013, to August 30, 2022. Study Design:

The study included all cases of female genital tuberculosis (FGTB) diagnosed through histopathology among gynecological specimens received by the Department of Pathology during the study period. Out of the 12,971 gynecological specimens examined, a total of 122 patients with a histopathological diagnosis of FGTB were identified from the department’s archives. Fourteen cases out of the 122 were excluded from the clinicopathologic analysis due to incomplete clinical data.

The evaluation of these patients and collection of their specimens were performed by multiple gynecologists using various gynecologic procedures such as cervical biopsy, vulvar punch biopsy, laparotomy biopsy, salpingectomy, salpingo-oophorectomy, total abdominal hysterectomy with salpingectomy, total abdominal hysterectomy with salpingo-oophorectomy, and total abdominal hysterectomy with salpingo-oophorectomy with colectomy.

### Inclusion criteria

All cases with a histopathological diagnosis of female genital tuberculosis.

### Exclusion criteria

Female genital TB cases with incomplete clinical data.

### Data collection and analysis

Clinical data for the eligible patients were collected from request forms and medical charts. Sixty-one tissue blocks that could be retrieved from the department’s archives were sent to the Armauer Hansen Research Institute (AHRI) for acid-fast bacilli (AFB) staining. The histological findings of FGTB at different sites, their correlation with clinical features, and the results of AFB staining were analyzed using the Statistical Package for the Social Sciences (SPSS).

### Operational definitions

Extragenital TB: In this study, extragenital tuberculosis (EGTB) was defined as tuberculosis infection affecting organs beyond the genital tract, including lymph nodes, bones, joints, and other body parts. The diagnosis of EGTB was based on organ-specific biopsy or cytology, as well as radiological and intraoperative findings that indicated the presence of tuberculosis.

Histopathologic Pattern: Histopathologic patterns were determined by staging granulomas according to their developmental stages, using a histopathological staging of *mycobacterial *granulomas that has been previously described (Table 1) [11].

**Table 1:** Histopathological staging of granulomas

Granuloma	Appearance	Necrosis
Stage 1	Irregular clusters of epithelioid cells interspersed with lymphocytes	Absent
Stage 2	Epithelioid cell aggregates with well-defined borders	Absent or minimal necrotic areas
Stage 3	Epithelioid cell aggregates with well-defined borders	Limited areas of central caseous necrosis
Stage 4	Multicentric epithelioid cell clusters with irregular borders	Extensive areas of caseous necrosis

## Results

### Prevalence of female genital tuberculosis (FGTB)

Over 10 years (January 2013-August 2022), FGTB was diagnosed in 0.94% (122) of the 12,971 biopsy specimens of female genital organs examined. The cases were distributed among various hospitals and health centers. The prevalence of FGTB varied from year to year, with the highest prevalence (1.8%) observed in 2013 and the lowest prevalence (0.32%) recorded in 2019 (Table 2).

**Table 2:** The annual prevalence of FGTB among gynecological specimens received inthe Department of Pathology between 2013 and 2022

Year	2013	2014	2015	2016	2017	2018	2019	2020	2021	2022	2013–2022
Total number of gynecology biopsies	1,105	1,131	1,124	1,278	1,297	1,356	1,582	1,345	1,616	1,137	12,971
FGTB cases Number(percentage)	20 (1.8%)	5 (0.44%)	11 (0.98%)	15 (1.17%)	12 (0.93%)	21 (1.55%)	5 (0.32%)	13 (0.97%)	9 (0.56%)	11 (0.97%)	122 (0.94%)

FGTB, female genital tuberculosis

### Description of clinicopathological findings

The study included women aged 16 to 78 years, with a mean age of 32.4 and a median age of 29. The distribution of age groups was as follows: 4.6% were under 20, 46.3% were between 20 and 29, 28.7% were between 30 and 39, 11.1% were between 40 and 49, and 9.3% were over 50.

The majority of women in the study (62%) were nulliparous, 27.8% were multiparous, and 10.2% were primiparous. Among the participants, 6.5% had HIV coinfection, and 15.7% had extragenital involvement. Peritoneal involvement was found in 58.8% of cases, gastrointestinal tract involvement in 35.3%, lung involvement in 29.4%, liver involvement in 17.6%, urinary tract involvement in 5.9%, and lymph node involvement in 5.9%.

The most common presentation was a menstrual disturbance, observed in 46.3% of cases. Amenorrhea was seen in 66%, and menometrorrhagia in 34%. Abdominal pain was the second most common presentation, seen in 34.3%, followed by infertility in 32.4%, and abdominopelvic mass in 29.6%. Other symptoms observed included vaginal discharge in 14.8%, extragenital symptoms in 14.8% (with weight loss in 7.4%, fever in 4.6%, appetite loss in 4.6%, cough in 3.7%, and night sweats in 2.8%), postmenopausal bleeding in 3.7%, dyspareunia in 1.9%, vulvar lesion in 1.9%, and postcoital bleeding in 0.9%. Ectopic pregnancy was observed in 1.9%, and early pregnancy loss in 0.9%. Additionally, 3.7% of the women were discovered incidentally, 0.9% during cervical cancer screening, and 2.8% after myoma surgery. Due to the presence of multiple signs and symptoms in women, the individual percentages do not add up to 100% (Table 3).

**Table 3:** Clinical presentations of patients with FGTB

Clinical presentation	Number (%)
Menstrual disturbance	50(46.3%)
Abdominopelvic pain	37(34.3%)
Infertility	35(32.4%)
Abdominopelvic mass	32(29.6%)
Vaginal discharge	16(14.8%)
Extragenital symptoms	16(14.8%)
Postmenopausal bleeding	4(3.7%)
Incidental/Asymptomatic	4(3.7%)
Dyspareunia	2(1.9%)
Vulvar lesion	2(1.9%)
Ectopic pregnancy	2(1.9%)
Post-coital bleeding	1(0.9%)
Early pregnancy loss	1(0.9%)

Among women with cervical TB, common symptoms included menstrual disturbance (50%), vaginal discharge (50%), abdominopelvic pain (37.5%), infertility (31.3%), extragenital symptoms (12.5%), postmenopausal bleeding (6.3%), and dyspareunia (6.3%). Additionally, a subset of patients (6.3%) were asymptomatic and did not show any symptoms. Only one asymptomatic patient with cervical tuberculosis had a recorded Pap smear result, which showed atypical squamous cells of undetermined significance (ASCUS). Subsequently, a cervical punch biopsy was performed, confirming tuberculosis in the tissue sample.

In our study, the diagnosis of FGTB in 108 patients relied on histopathological examination of tissues obtained through various methods: 52.8% through endometrial curettage, 13.9% through cervical punch biopsy, 1.9% through vulvar punch biopsy, 12% through laparotomy biopsy of peritoneum or adnexal mass, 4.6% through salpingectomy, 7.4% through salpingo-oophorectomy (SO), 2.8% through total abdominal hysterectomy (TAH) with salpingectomy, 3.7% through TAH with SO, and 0.9% through TAH with SO and colectomy (Table 4).

**Table 4:** Procedures performed on patients with FGTB to acquire tissue for histopathological examination

Specimen	Number(Percent)
Endometrial curettage	57(52.8%)
Cervical punch	15(13.9%)
Laparotomy biopsy	13(12%)
SO	8(7.4%)
Salpingectomy	5(4.6%)
TAH + SO	4(3.7%)
TAH + Salpingectomy	3(2.8%)
Vulvar punch	2(1.9%)
TAH + SO + Colectomy	1(0.9%)
Total	108(100%)

SO, salpingo-oophorectomy; TAH, total abdominal hysterectomy

Table 5 presents the clinical and histopathological diagnoses of women who underwent resection. In our study, two women underwent salpingectomy for ectopic pregnancy, and three women underwent total abdominal hysterectomy with salpingectomy to remove a large myoma, which led to the diagnosis of tuberculosis salpingitis. Among women who had salpingo-oophorectomy, three had concurrent benign ovarian cysts, and one woman with an excisional biopsy also had a benign cyst. Four women underwent total abdominal hysterectomy with salpingo-oophorectomy, and one woman had a total abdominal hysterectomy with salpingo-oophorectomy and colectomy due to suspected malignancy based on clinical and radiologic evidence. Histopathological findings confirmed ovarian TB with peritoneal involvement in three patients, including one case with a concomitant benign ovarian cyst, and endometrial TB in the other two cases.

**Table 5:** Clinicalandhistopathological diagnosis of patients with FGTB who underwent resection

Type of Procedure	Percent (Number)	Clinical diagnosis	Histopathological diagnosis
SO	7.4% (8)	2(Pyogenic abscess R/O TB) 6(Ovarian tumor R/O TB)	TB + 3 (benign ovarian cyst (2 serous &1 Benign))
Salpingectomy	4.6% (5)	2(Ectopic pregnancy) 3 (Infertility 2^0^ R/O TB)	TB salpingitis + 2(Ectopic pregnancy)
TAH + Salpingectomy	2.8% (3)	Myoma	Myoma + TB salpingitis
TAH + SO	3.7% (4)	2(Advanced ovarian tumor)	Ovarian TB with peritoneal involvement + serous cyst adenoma (1)
		2(Endometrial neoplasm R/O TB)	Endometrial TB
TAH + SO + Colectomy	0.9% (1)	Advanced ovarian tumor	Ovarian TB with peritoneal and colonic involvement

SO, salpingo-oophorectomy; TAH, total abdominal hysterectomy; TB, tuberculosis; R/O, rule out

In our study, the most commonly involved site was the endometrium (55.6%), followed by the fallopian tube (23.1%), ovary (14.8%), cervix (14.8%), and vulva (1.9%). Multifocal tuberculosis was observed in 9.3% of cases, resulting in the percentage not totaling 100% (Table 6). Among these cases, 7.4% had tuberculosis affecting both the fallopian tube and ovary, 0.93% had tuberculosis affecting the endometrium and cervix, and another 0.93% had tuberculosis involving the fallopian tube, endometrium, and cervix simultaneously.

**Table 6:** Female reproductive organs involved in TB

Organ	Number(Percent)
Endometrium	60(55.6%)
Fallopian tube	25(23.1%)
Ovary	16(14.8%)
Cervix	16(14.8%)
Vulva	2(1.9%)

**Fig. 1:**
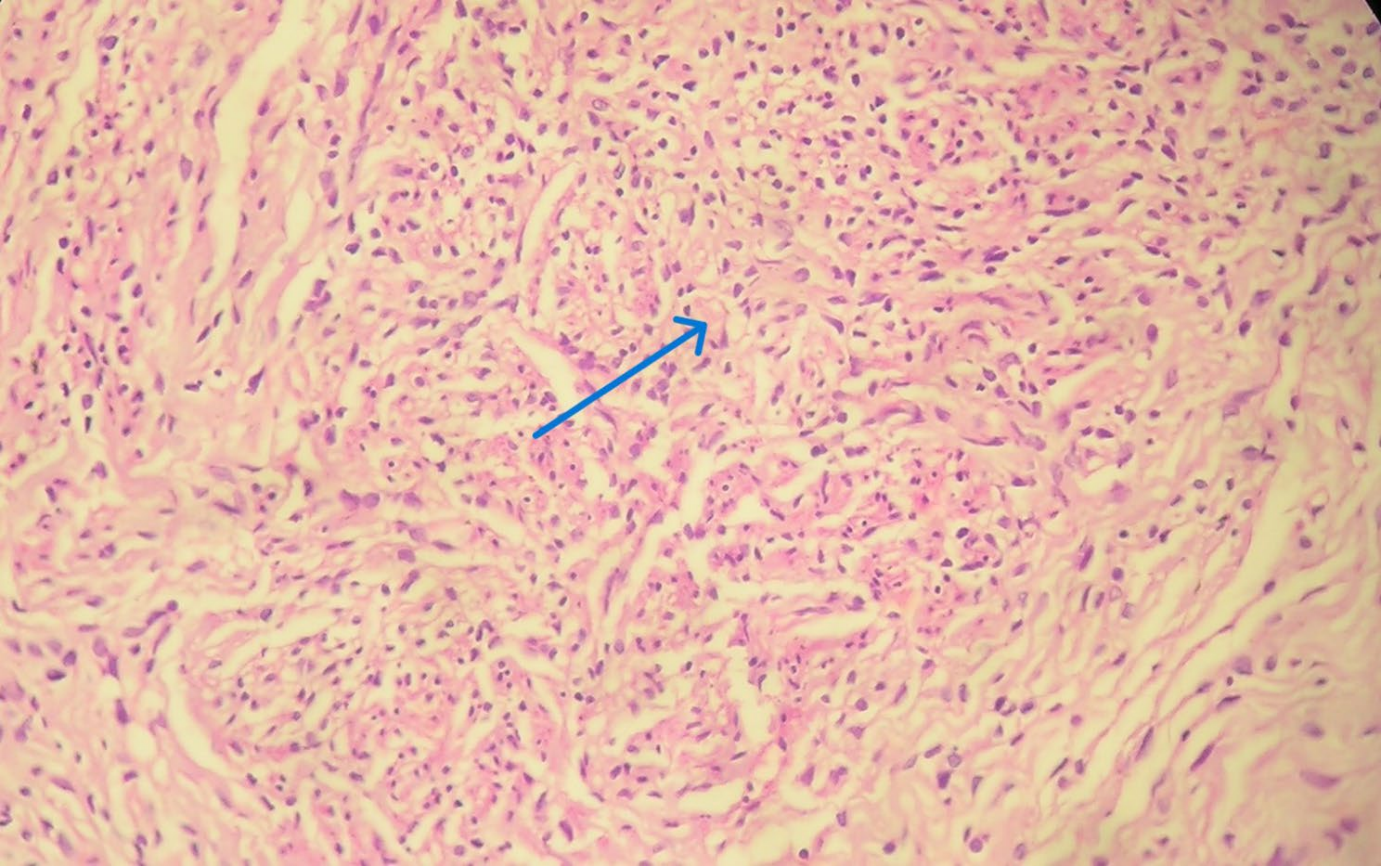
Photomicrograph showing tissue with stage 1 granulomas. The arrow shows irregular clusters of epithelioid cells

**Fig. 2:**
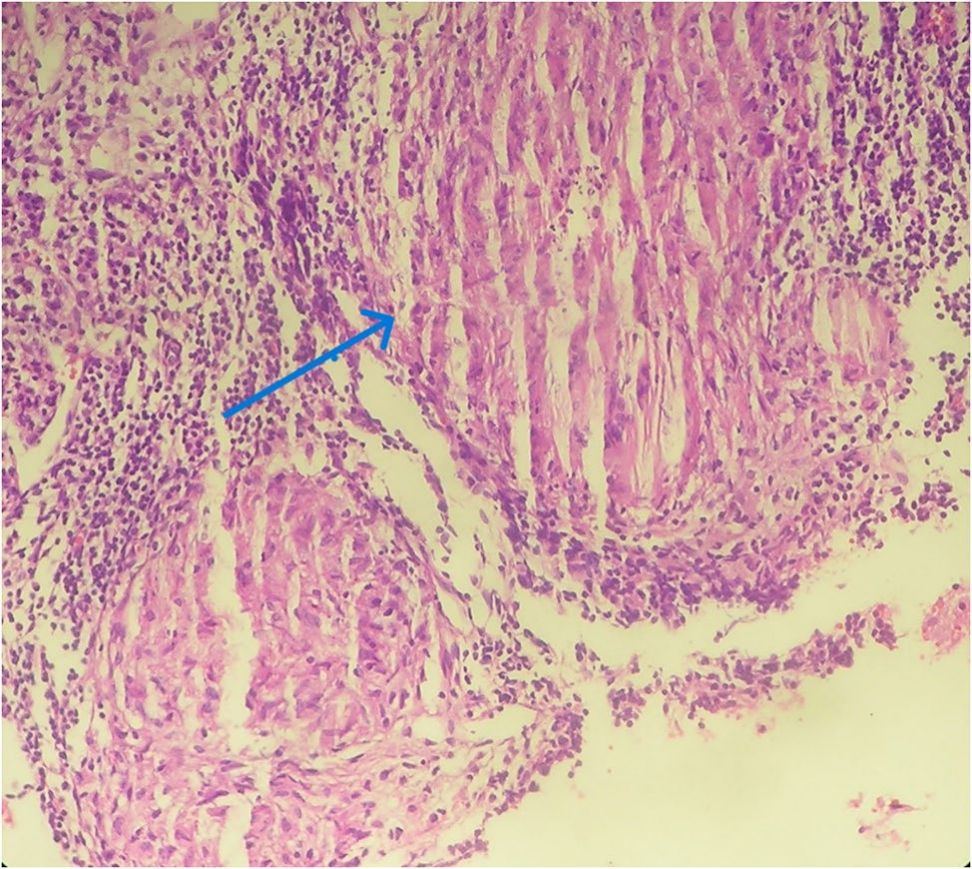
Photomicrograph showing tissue with stage 2 granulomas. The arrow shows epithelioid cell aggregates with well-defined borders

**Fig. 3:**
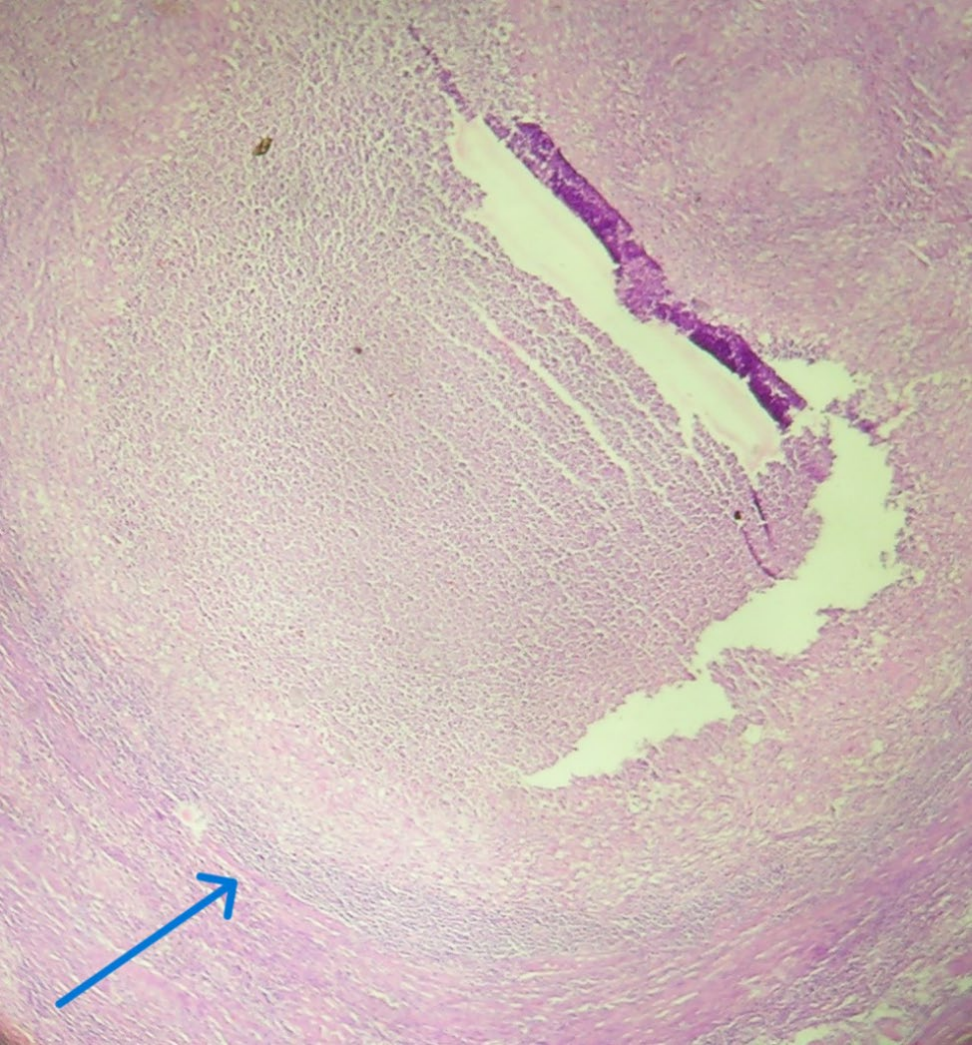
Photomicrograph showing tissue with stage 3 granulomas. The arrow shows epithelioid cell aggregates with well-defined borders and limited central caseous necrosis

**Fig. 4:**
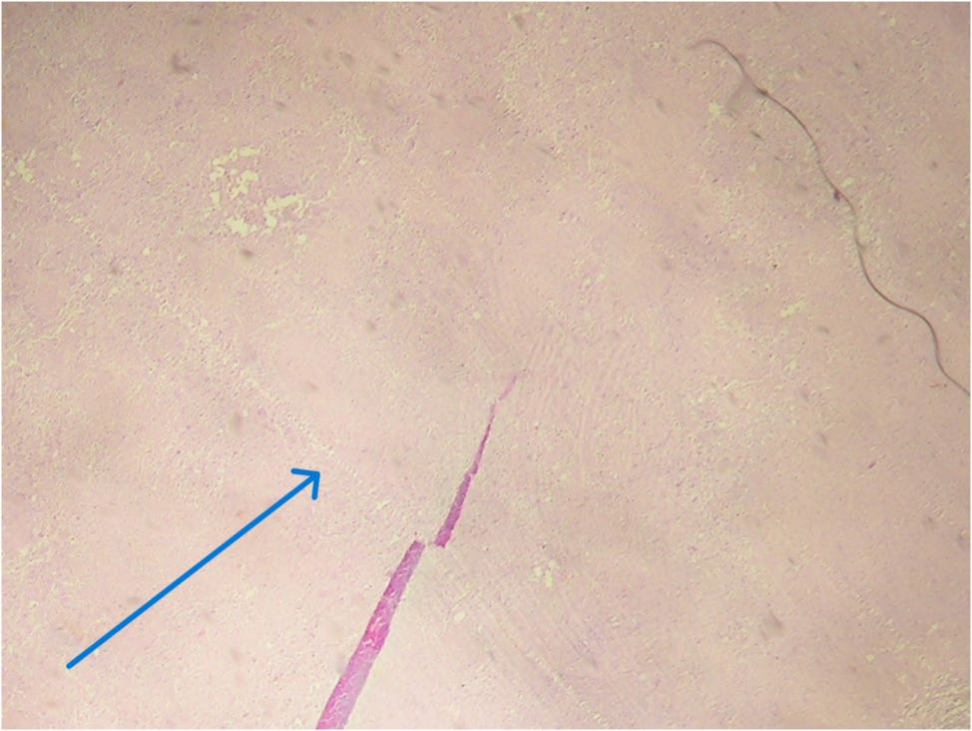
Photomicrograph showing tissue with stage 4 granulomas. The arrow shows extensive areas of caseous necrosis

Histopathologic examination revealed four major types of granulomas, classified as stages 1 to 4. In the ovary, the majority of granulomas were well-formed to multicentric, with limited to extensive caseous necrosis. 87.5% were classified as stage 3 or 4, while 12.5% were classified as stages 1 to 2 (Figs. 1, 2, 3, 4 and Table 7). Similarly, 68% of the fallopian tube granulomas were classified as stage 3 or 4, while 32% were classified as stage 1 to 2. In contrast, cervix granulomas mainly appeared as vague clusters of epithelioid cells or had well-defined borders with minimal or no necrosis. 87.6% of cervix granulomas were classified as stage 1 to 2, while 12.5% were stage 3 or 4. All vulva granulomas were classified as stages 1 to 2. In the endometrium, 53.3% of granulomas were stage 1 to 2 and 46.7% were stage 3 or 4. Among women with stage 3 to 4 granulomas, six had regular cycles, five were postmenopausal, thirteen were amenorrheic, and four had menometrorrhagia. Among those with stage 1 to 2 granulomas, ten had regular cycles, one was postmenopausal, nine were amenorrheic, nine had menometrorrhagia, and three had postmenopausal bleeding.

**Table 7:** Histopathologic patterns of FGTB

	Stage of granuloma				
**Organ involved**		**Stage 1**	**Stage 2**	**Stage 3**	**Stage 4**
	Ovary	0(0%)	2(12.5%)	8(50%)	6(37.5%)
	Fallopian tube	0(0%)	8(32%)	8(32%)	9(36%)
	Endometrium	3(5%)	29(48.3%)	22(36.7%)	6(10%)
	Cervix	3(18.8%)	11(68.8%)	2(12.5%)	0(0%)
	Vulva	0(0%)	2(100%)	0(0%)	0(0%)

Among the 108 women in the study, seven had HIV coinfection. Among those coinfected with TB/HIV, three had TB endometritis, two had TB cervicitis, one had TB vulvitis, and one had TB salpingitis. Microscopic examination revealed scattered epithelioid cells (stage 1 granulomas) in one case, well-formed granulomas without necrosis (stage 2 granulomas) in four cases, and extensive caseation with granulomas (stage 4 granulomas) in two cases.

Acid-fast bacilli were detected in 42.6% of the 61 genital tract tissues with TB histopathology using both ZN and auramine staining. Among these cases, 17 showed AFB presence with both stains, 4 cases with ZN stain only, and 5 cases with auramine stain alone. However, AFB was not found in the four tissues with HIV coinfection using both auramine and ZN stains. In terms of specific organs, AFB was isolated in 75% of the ovaries (3 out of 4), 50% of the fallopian tubes (7 out of 14), 38.7% of the endometrium (12 out of 31), and 33.3% of the cervix (4 out of 12) (Table 8). AFB was detected in 35.3% of tissues with stage 1–2 granulomas and 51.9% of tissues with stage 3–4 granulomas. Pearson correlation analysis shows a weak positive correlation (0.204) between AFB and the stage of granuloma. This correlation is not statistically significant (*p* = 0.114) based on a sample size of 61.

**Table 8:** TB at different anatomic sites with the corresponding AFB staining patterns

Site/Organ	AFB present in tissue section (Z-*N* or auramine stain)
Ovary	75% (3/4)
Fallopian tube	50% (7/14)
Endometrium	38.7% (12/31)
Cervix	33.3% (4/12)
Total	42.6% (26/61)

AFB, acid fast bacilli

## Discussion

This study analyzed 122 cases of Female Genital Tuberculosis (FGTB) at AAU in Ethiopia. The diagnosis of FGTB presents challenges and requires clinical suspicion and diagnostic tools. The objective of the study is to assess the prevalence, trends, and clinical/histopathologic patterns of FGTB in gynecology specimens received by the pathology department.

The study found that TB was present in 0.94% of female genital tract specimens analyzed over the past ten years. The annual percentage has been declining, with the highest occurrence in 2013. Similarly, a previous study conducted in the same department in 1999 reported a prevalence of 1.38% [9]. These decreasing trends align with the national decline in TB incidence since 1995 [12]. In contrast, in New Delhi, India, TB endometritis was identified in 2.3% of nonpregnant endometrial samples [13]. Likewise, a study in the TASH gynecology department in 2018 reported varying prevalence rates of endometrial TB using different diagnostic methods: 4.6% using PCR, 2.6% using culture, and only 1.3% using histological examination [14].

Furthermore, a study by Radhika et al. compared different diagnostic methods for female genital tuberculosis, revealing that combining PCR with BACTEC increased sensitivity to 52% [15]. Similarly, Sethi et al. emphasized the inadequacy of relying on a single test for diagnosing genital tuberculosis and stressed the importance of clinical evaluation in conjunction with a combination of conventional and newer diagnostic methods [16].

Considering the relatively low proportion of TB cases observed in this study, it is reasonable to suggest that the absence of alternative diagnostic methods, such as culture and more sensitive molecular techniques, may have resulted in an underestimation of TB prevalence. Therefore, future research should incorporate these advanced modalities to improve the accuracy of diagnosing female genital tuberculosis.

In this study, most of the patients were between the ages of 20 and 39, accounting for 75% of the cases. A total of 9.3% were over the age of 50, with no cases documented under the age of 15. This finding is consistent with the study conducted here in Ethiopia by Abebe et al., which shows that the majority of cases (83.18%) occurred between the ages of 20 and 40 [9]. Similarly, in a study conducted in Pakistan by Qureshi et al., the majority of cases (75%) were between the ages of 20 and 45 [17]. Furthermore, in a study performed in Uganda by Othieno et al., the most common age group was 21–30 years, accounting for 48.7% of the cases [18]. The young age observed in these populations’ patients could be explained by early marriages and pregnancies, as well as the overall young age of the population. In addition, the atrophic endometrium in elderly women is not a favorable environment for the development of *mycobacterium TB*, which explains the rarity of tuberculosis in postmenopausal patients [19]. Our finding is different from the study in Sweden by Falk et al., which showed that GTB was more common after menopause [10].

In this study, the most common presentations were menstrual disturbance (46.3%), abdominopelvic pain (34.3%), and infertility (32.4%). In the study by Abebe et al., abnormal uterine bleeding (45.5%) and infertility (36%) were the most common presentations [9]. Similarly, in the study by Othieno et al., the most common presentations were abnormal uterine bleeding (35.9%), infertility (28.2%), and abdominal distention (28%) [18]. However, in most studies, the most common complaint in women with GTB is infertility, followed by abdominopelvic pain, and abnormal uterine bleeding. In a study in India by Mondal et al., 65–70% of patients presented with infertility, 50–55% with pelvic/abdominal pain, and 20–25% with menstrual disturbances [20]. Similarly, Qureshi et al. observed that infertility was the most common presenting symptom (42.5%), followed by abdominal pain (42%) [21]. Because the patients’ marital status was not recorded in this study, it was difficult to pinpoint a cause for the documented low infertility rate compared to most studies. Nevertheless, it is generally agreed that menstrual disorder occurs in approximately 40–50% of women who suffer from GTB, which is consistent with our study.

In the present study, three patients with GTB were misdiagnosed as having malignant disease, resulting in aggressive surgical management. Two patients underwent TAH with SO, and one patient underwent TAH with SO and colectomy. On histopathology, they were found to have ovarian TB with peritoneal involvement and, in one case, a concomitant benign ovarian cyst. Similar to our study, retrospective studies conducted in Malaysia and Turkey found that patients with tubo-ovarian TB associated with peritoneal involvement (ascites) and a frequently associated high level of serum CA-125 are frequently misdiagnosed with ovarian carcinoma and are subjected to unnecessary and aggressive surgery [22, 23]. Furthermore, case reports by Lobo et al. from Singapore and Yang et al. from China documented the synchronous occurrence of benign cystic neoplasm and ovarian TB and the diagnostic difficulty it poses [24, 25]. In the literature thus far, few cases of ovarian TB and coexisting benign cystic neoplasm of the ovary have been reported, and all cases were regarded as mere coincidences [24–26]. In this study, which was conducted in a country with a high TB prevalence, 31.3% of ovarian TB patients had concurrent benign cystic neoplasms. Hence, it is advisable to carry out more research to examine this relationship because it may be more than just a coincidence.

In this study, the endometrium was the most frequently affected organ, accounting for 55.6% of the cases, followed by the fallopian tube (23.1%), the ovary (14.8%), the cervix (14.8%), and the vulva (1.9%). Similar results to ours were also observed in studies conducted by Abebe et al., Turkmen et al., & Mondal et al. [9, 20, 27]. In contrast, the study by Nogales et al. found that the fallopian tube was the most frequently affected [17]. When FGT occurs, the fallopian tube is involved in nearly all patients, and involvement of the endometrium is usually secondary to tubal disease [19]. Thus, the fact that tubal involvement was less common in our study than endometrial involvement may be attributed to sampling bias due to the easy accessibility of the endometrium for biopsy, and the majority of the specimens received in our department were endometrial biopsies. In the endometrium, 46.7% of cases showed well-formed to multicentric granulomas with varying degrees of caseous necrosis, whereas Mondal et al. found endometrial caseation in only 2.6% of cases [20]. The histopathology of FGTB typically resembles tuberculosis in other tissues, but advanced-stage caseation, fibrosis, and calcification are uncommon in the endometrium during the reproductive period due to regular cyclical shedding [28]. Progesterone has a “flaring” effect on the endometrial tuberculosis located at the basal layer. The highest levels of progesterone occur in the middle of the second half of the menstrual cycle. Therefore, the optimal time to perform an endometrial biopsy to assess would be in the middle of the luteal phase.

The higher percentage of caseation observed in our study may be due to the presence of amenorrheic or postmenopausal women among those with caseation. However, in line with most studies, the majority of patients in our study (53.3%) had early-stage immature granulomas. Therefore, it is advisable to conduct biopsies just before menstruation or during the late secretory phase.

In our study, AFB was detected in 42.6% of the FGT tissues with a histopathological diagnosis of TB. In a study conducted by Eshete in Ethiopia on 60 lymph node tissues with a histopathological diagnosis of TB, AFB was detected in 61.7% [29]. Similarly, in a study performed by Krishnaswanni in India on 128 lymph node tissues and by Rasool in Pakistan on 50 lymph node tissues, both with a histopathological diagnosis of TB, AFB was detected in 79% and 56% of the cases, respectively [30, 31]. In another study conducted in England by Greenwood and Fox on 70 cases with a histopathological diagnosis of TB from various anatomical sites (including 21 lymph nodes, 9 kidneys, 6 gastrointestinal tracts, 5 epididymis, 4 livers, 4 omenta, 3 synoviums, 1 brain, 1 vagina, 1 heart, 1 spleen, and 1 skin), AFB was detected in 60% [32]. Similarly, a study conducted in America by Koch et al. on 136 tissues (100 from the lung, 28 from lymph nodes, 5 from the spleen, and 1 each from the heart, liver, and bone marrow) from different anatomic sites found that 34.5% were positive for AFB [33].

The relatively lower rate of AFB detection in our study compared to other studies may reflect the paucibacillary nature of FGTB. Consistent with the paucibacillary nature of the disease in our study, other studies have found a low overall detection rate of FGTB by AFB staining. According to Malhotra et al., the AFB detection rate was 2.7%, 1.6% according to Bhanu et al., and 8.3%according to Thangappah [34–36]. The higher detection rate of AFB in our study compared to these studies is most likely because the AFB stain was performed on tissues with a histopathological diagnosis of TB, unlike the other studies that were conducted on clinically suspected tissues. Furthermore, in the other studies, only the ZN stain was used, whereas in this study, both ZN and auramine stain were used, which may have contributed to the observed higher rate.

In this study, auramine detected AFB in 22 tissues, and ZN detected AFB in 21 tissues (17 cases stained positive with both ZN and auramine, 4 cases with ZN only, and 5 cases with auramine stain only). This finding supports the higher sensitivity of the auramine stain, as documented in the literature. A study conducted in India by Krishnaswanni compared ZN and fluorescent techniques for AFB demonstration on 128 lymph node tissues with a TB histopathology diagnosis found 71.1% positive for AFB after ZN staining and 79.7% positive for AFB on fluorescent techniques [30]. Similarly, a study conducted in England by Greenwood and Fox on 70 tissues from various anatomical sites with a TB histopathology diagnosis found 47.1% positive for AFB with the ZN staining technique and 60.0% positive for AFB with the fluorescent technique [32]. A study conducted in Pakistan by Rasool on 50 lymph node tissues with a TB histopathology diagnosis revealed that 54% were positive for AFB after ZN staining and 56% were positive for AFB after fluorescence staining [31]. Bacteria are easier to visualize with the auramine stain, so tissues that stain positive with ZN also stain positive with auramine. The four tissues in our study that stained positive for ZN but not for auramine could be attributed to technical issues, which might have contributed to the slight difference in auramine and ZN positivity compared to the other studies.

In this study, the ovary had the highest rate of AFB detection, followed by the fallopian tube, endometrium, and cervix. AFB was detected in 35.3% of tissues with stage 1–2 granulomas and 51.9% of tissues with stage 3–4 granulomas. Therefore, the relationship between AFB positivity and granuloma stage was not statistically significant, with a p-value of 0.1. Although we observed a trend toward higher AFB detection in stage 3–4 granulomas, further studies with larger sample sizes are needed to confirm this finding. Among the 7 TB/HIV coinfected women, 1 had stage-1 granulomas, 2 had stage-4 granulomas, and the remaining 4 had stage-2 granulomas. AFB was not detected in the four tissues that were examined. However, it is important to note that the histologic features of TB granulomas can vary depending on the patient’s immune status. Therefore, conducting a larger study with a stratified study population based on immunosuppression levels would provide a better characterization of the histologic features of FGTB with HIV coinfection.

The strength of this study, compared to other similar studies, is the inclusion of patients over ten years resulting in a relatively large sample size, which increases the study’s importance. However, a limitation of this study is that definitive evidence of *mycobacterium TB *infection using more sensitive tests such as PCR/culture was not performed due to unavailability issues.

## Conclusion

This study assessed the prevalence, trends, and characteristics of FGTB in gynecological specimens. Despite a decline, FGTB remains a significant health problem, which may be underestimated due to reliance on histology for diagnosis. GTB should be considered in women experiencing menstrual disturbances, abdominopelvic pain, and infertility. TB should be included as a potential differential diagnosis in women, especially those in high TB prevalence populations or of reproductive age, who present with abdominopelvic mass and ascites. This can help prevent unnecessary aggressive surgery. A diagnostic workup is advised for suspected GTB cases. The endometrium is the most frequently involved organ, followed by the fallopian tube, ovary, cervix, and vulva. Due to the paucibacillary nature and cyclical shedding of the endometrium, TB endometritis often shows early-stage granulomas and low AFB detection.

## Data Availability

The dataset supporting the findings of this study was generated at TASH and AHRI and is available from the corresponding author on request.

## Abbreviations

FGTB: Female Genital Tuberculosis
AFB: Acid-fast Bacilli
CHS: College of Health Science
TASH: Tikur Anbessa Specialized Hospital
AAU: Addis Ababa University
TB: Tuberculosis
PCR: Polymerase Chain Reaction
WHO: World Health Organization
EPTB: Extrapulmonary Tuberculosis
GTB: Genital Tuberculosis
HIV: Human Immunodeficiency Virus
COVID19: Coronavirus Disease 2019
CHS-IRB: Institutional Review Board of the College of Health Science
AHRI: Armauer Hansen Research Institute
SPSS: Statistical Package for the Social Science
TAH: Total Abdominal Hysterectomy
SO: Salpingo-oophorectomy
ZN: Ziehl-Nelsen
FGT: Female Genital Tract
